# Efficacy of Electrical Stimulation for Spinal Fusion: A Systematic Review and Meta-Analysis of Randomized Controlled Trials

**DOI:** 10.1038/s41598-020-61266-x

**Published:** 2020-03-12

**Authors:** Shakib Akhter, Abdul Rehman Qureshi, Idris Aleem, Hussein Ali El-Khechen, Shadman Khan, Omaike Sikder, Moin Khan, Mohit Bhandari, Ilyas Aleem

**Affiliations:** 1 Department of Health Research Methods, Evidence, and Impact, McMaster University, Hamilton, Canada; 2 Department of Orthopaedic Surgery, McMaster University, Hamilton, Canada; 3 OrthoEvidence, Burlington, Ontario Canada; 4 Department of Orthopaedic Surgery, University of Michigan, Ann Arbor, MI USA; 5 North Inc, Kitchener, ON Canada

**Keywords:** Fracture repair, Orthopaedics, Epidemiology

## Abstract

Spinal fusion is one of the most common procedures performed in spine surgery. As rates of spinal fusion continue to increase, rates of complications such as nonunions continue to increase as well. Current evidence supporting the use of electrical stimulation to promote fusion is inconclusive. This review aimed to determine if postoperative electrical stimulation is more efficacious than no stimulation or placebo in promoting radiographic fusion in patients undergoing spinal fusion. We searched the Cochrane Central Register of Controlled Trials (CENTRAL), EMBASE, CINAHL and MEDLINE from date of inception to current. Ongoing clinical trials were also identified and reference lists of included studies were manually searched for relevant articles. Two reviewers independently screened studies, extracted data, and assessed risk of bias. Data were pooled using the Mantel-Haenszel method. Trialists were contacted for any missing or incomplete data. Of 1184 articles screened, 7 studies were eligible for final inclusion (n = 941). A total of 487 patients received postoperative electrical stimulation and 454 patients received control or sham stimulation. All evidence was of moderate quality. Electrical stimulation (pulsed electromagnetic fields, direct current, and capacitive coupling) increased the odds of a successful fusion by 2.5-fold relative to control (OR = 2.53, 95% CI 1.86 to 3.43, p < 0.00001). A test for subgroup interaction by stimulation type, smoking status, and number of levels fused was not significant (p = 0.93, p = 0.82 and p = 0.65, respectively). This systematic review and meta-analysis found moderate-quality evidence supporting the use of postoperative electrical stimulation as an adjunct to spinal fusion surgery. Patients treated with electrical stimulation have significantly greater rates of successful fusion. The level of evidence for this study is therapeutic level I.

## Introduction

Back pain is the most common presentation of numerous spinal pathologies, significantly affecting patient health and quality of life^1,2^. It is estimated that one in five patients with back pain will require surgical intervention, most commonly spinal fusion^1^. Spinal pathologies such as spinal stenosis with instability, spondylolisthesis, and spinal deformity are common indications for spinal fusion^2^. Direct and indirect costs of spinal fusion are estimated to be more than 9 billion US dollars annually^1,3^. Spinal fusion is becoming increasingly common given the aging population, as in the United States alone spinal fusion incidence for degenerative indications exponentially increased from 7.5 per 100,000 to 17.8 per 100,000 between 2000 and 2009, respectively^4^. While the procedure can significantly improve quality of life, outcomes may be negatively impacted by complications such as nonunion, pseudarthrosis, and hardware failure^5,6^. The rate of nonunion is estimated to be between 25%-81%, indicating a compelling challenge in obtaining successful spinal fusion^7^.

A number of adjunctive therapies such as biological agents or electrical stimulation have been advocated to promote spinal fusion^8^. Electrical stimulation has been suggested to improve fusion rates through the direct and indirect upregulation of bone morphogenic proteins, stimulating bone formation and remodeling^8–10^. Three types of electrical stimulation have been approved by the Food and Drug Administration: (1) pulsed electromagnetic fields (PEMF); (2) direct current electrical stimulation (DC); and (3) capacitive coupling (CC)^11^. DC stimulation involves surgical implantation of a cathode at the fusion site and an anode within the soft tissue, providing constant direct stimulation to the site^12^. In contrast, PEMF and CC are non-invasive techniques involving electrodes or a fitted coil placed over the skin, respectively^12,13^.

Following Dwyer’s initial report on the clinical utility of electrical stimulation for spinal fusion in 1974^14^, a number of studies have since evaluated its efficacy on clinical and radiographic outcomes after spinal fusion^13,15–21^. Within these trials, methodological flaws have led to inconclusive and conflicting findings^13,15–21^. Current systematic reviews and meta-analyses evaluating spinal fusion are limited due to methodological flaws or limited inclusion criteria^22–26^. The need to systematically evaluate the effect of electrical stimulation with respect to spinal fusion is required to provide clinicians with a current best estimate of efficacy^13,15–17,21,22^. We therefore sought to determine the efficacy of postoperative electrical stimulation on radiographic fusion rates at a minimum 1-year follow-up in adult patients following spinal fusion.

## Methods

We conducted this study as per the guidelines outlined by the Cochrane Handbook for Systematic Reviews of Interventions^21^ as well as the Preferred Reporting Items for Systematic Reviews and Meta-Analyses (PRISMA) statement^27^.

### Identification of studies

We searched the Cochrane Central Register of Controlled Trials (CENTRAL) to Jan 28, 2018, EMBASE (OVID- 1980 to Jan 28, 2018), CINAHL (1982 to Jan 28, 2018) and MEDLINE (OVID -1946 to Jan 28, 2018). To limit search findings to only randomized controlled trials, we combined the Cochrane highly sensitive search strategy (sensitivity-maximizing version) to our MEDLINE search. No limits to publication date or language were placed on the search^28^. Alterations were made to the Cochrane sensitivity maximizing search strategy to identify randomized trials in the CENTRAL, EMBASE, and CINAHL databases^29^. The search strategies for the databases are presented in Table 1. Ongoing clinical trials were identified using the World Health Organization’s International Clinical Trials Registry Platform (ICTRP) and the clinicaltrials.gov database. Reference lists of included studies were manually searched for relevant articles. Other relevant research available as grey literature was searched through the HLWIKI International database.

### Assessment of eligibility

Two review authors independently screened titles and abstracts for inclusion. A full text screening ensued to further elicit articles for inclusion by applying eligibility criteria to the methods section. Any disagreements were resolved through discussion. Agreement of reviewers’ assessment for study eligibility was calculated using Cohen’s kappa coefficient (κ), with κ ≥ 0.65 being considered adequate^30^.

Trials that included fracture cases or had a minimum follow-up of less than 1 year were excluded. The population of interest included individuals aged 18 or older undergoing spinal fusion surgery at any level (cervical, lumbar, thoracic) for any degenerative spinal pathology. Tumor and fracture cases were excluded. Randomization to post-operative electrical stimulation as an adjunct to spinal fusion was compared with no stimulation or placebo. The intervention group was organized by type of stimulation: pulsed electromagnetic fields, direct current electrical stimulation, and capacitive coupling. We included trials in which either of the three stimulation modalities were used, and were compared to no stimulation or placebo (sham stimulation).

All studies adhering to the following criteria were included:

1. randomized controlled trials.
2. comparing either DC, CC, or PEMF electrical stimulation to sham, placebo-controlled, or no stimulation as an adjunct to spinal fusion surgery.

### Data extraction and management

Two review authors independently extracted data from each study into a Microsoft Excel data form. Data included primary author’s last name, publication year, funding source, all outcomes reported and scales used, length of study and outcome follow-up, type of stimulation, and type of comparator (no stimulation or placebo). Furthermore, data for treatment and control groups were extracted in terms of sample size, age, gender, and missing or lost data. In the event where important data was unclear or missing, we attempted to contact study authors to retrieve such information.

### Assessment of risk of bias

The recommendations outlined in the *Cochrane Handbook for Systematic Reviews of Interventions* guided the assessment of risk of bias for all trials included in this review^31^. The assessment of risk of bias was conducted by two reviewers and the Cochrane software Review Manager 5 (RevMan) was used to compile our assessments^32^. The assessment domains included: random sequence generation (selection bias); allocation concealment (selection bias); blinding of participants and personnel (performance bias); blinding of outcome assessment (detection bias); incomplete outcome data (attrition bias); selective reporting (reporting bias). Each domain was judged as ‘low’, ‘high’, or ‘unclear’ and the reason for judgments is supported with direct evidence and interpretation from the trial publication (Table 2). The intraclass correlation coefficient (*r*) was used to calculated reviewer agreement for the risk of bias assessment.

### Statistical analyses

Dichotomous outcome of fusion was pooled via the Mantel-Haenszel method. We used Review Manager 5 to calculate the odds ratio (OR) and 95% confidence interval. All data analysis and presentation was performed using Review Manager 5. OR was selected as the measure of treatment effect considering the relative ease in interpretation. Heterogeneity was quantified using the I^2^ statistic from the Chi-squared test for heterogeneity. In accordance to the Cochrane Handbook, heterogeneity for I^2^ values between 30–60% may be moderate, 50–90% may be substantial, while 75–100% may be considerable^31^.

Alternatively, another approach to the analysis would be employing hierarchical testing coupled with a model-based regression analysis. Subgroups could have been prioritized by clinical importance, and if the higher priority subgroup’s effect is statistically insignificant, then the subgroup analyses with lower priority would not be tested. However, considering a test of interaction demonstrated that the difference in subgroup effects was statistically insignificant, the studies were instead pooled to estimate an overall effect”.

### Subgroup analysis and investigation of heterogeneity

Subgroup analyses investigating any differences in effect by type of stimulation, fusion level, and smoking status were preplanned. We were interested in exploring variations in effect between these population subgroups provided a higher rate of complications following spinal fusion are observed in patients who smoke or are elderly, which may influence the relative efficacy of electrical stimulation^15^. To explain any potential heterogeneity, we pre-specified a subgroup analysis of type of stimulation (pulsed electromagnetic fields, direct current electrical stimulation, and capacitive coupling). Trials with the same stimulation technique were pooled and analyzed separately from trials with other stimulation techniques. Tests for interaction were also performed for this subgroup using a chi-squared significance test^33^.

### Sensitivity analysis

A sensitivity analysis was conducted to explore the impact of incomplete outcome data. Trials with high risk of bias in the incomplete outcome data domain of the risk of bias assessment were excluded. A second sensitivity analysis considered the variability in evaluating fusion. Only trials in which outcome assessment was done by an independent blinded radiologist were included. Percentage changes in ORs between the sensitivity analysis and the main analysis were reported. They were calculated by dividing the ORs from the main analysis by the ORs from the sensitivity analysis, and the resulting fraction was converted to a percentage by subtracting by 1 and multiplying by 100.

### Assessment of the certainty of the evidence

We used the GRADE approach to assess the quality of evidence for the use of electrical stimulation^31^. The GRADE domain of likelihood of publication bias was assessed statistically and non-statistically using the guidelines outlined by Murad and colleagues on conducting GRADE for narrative reviews, considering a funnel plot was not produced as only 7 studies were included in the review^34,35^.

## Results

### Description of search results

**Figure 1:**
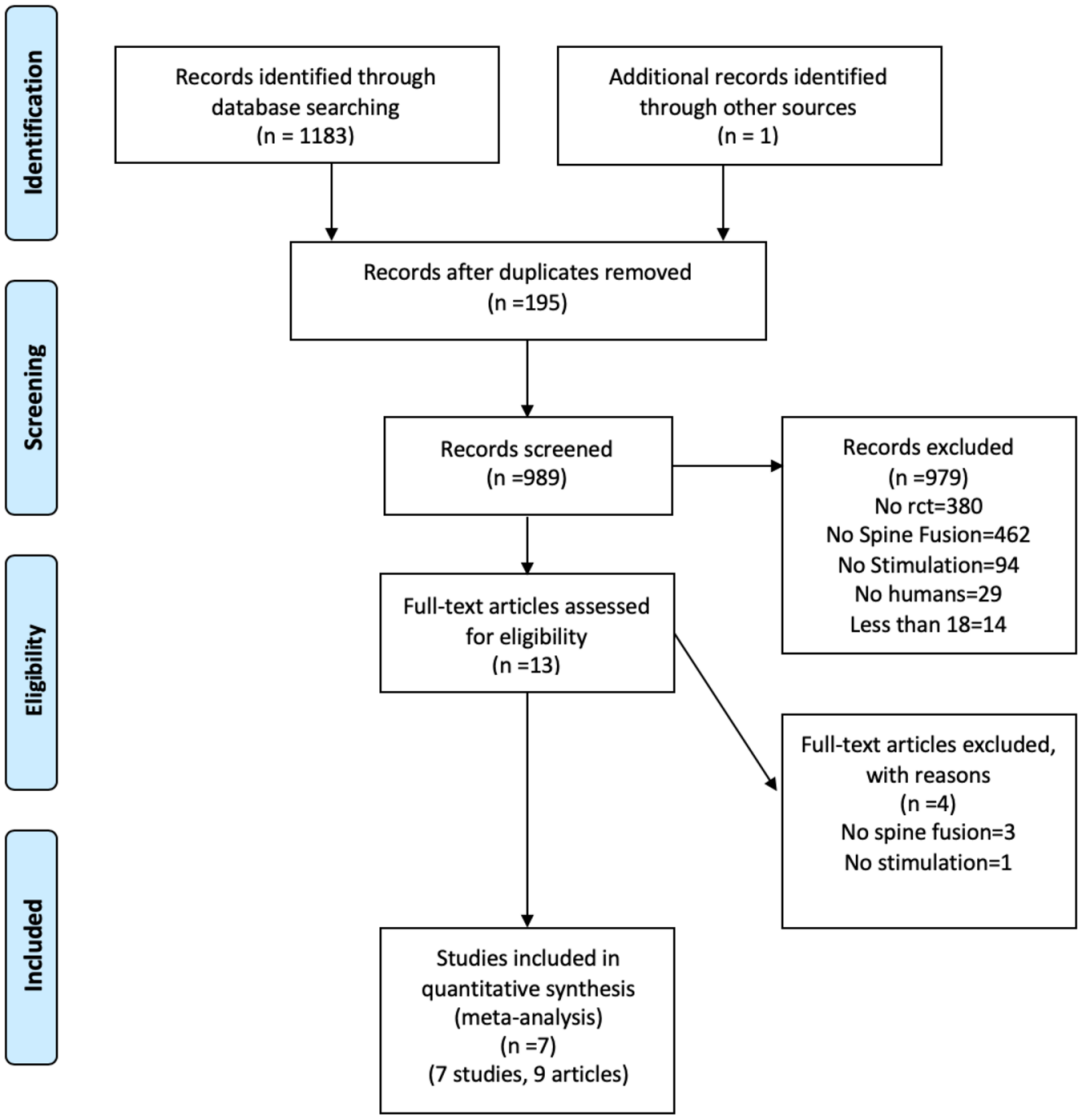
Risk of bias assessment.

Our search identified 1184 articles. After excluding 195 duplicates, a total of 989 titles and abstracts were screened and 9 articles (7 studies) were eligible for our systematic review and meta-analysis (Fig. 1). Thus, seven studies were included with a total of 941 patients, of which 487 patients received postoperative electrical stimulation and 454 patients received placebo or sham stimulation. No additional trials were identified from gray literature, ongoing trial registries, or conference proceedings. Agreement between the reviewers for study eligibility was moderately high (κ = 0.88, 95% CI: [0.81, 0.94], p < 0.0001).

### Study characteristics

Mean age of study participants was 51.2 and 49.9 years in the treatment and control arms, respectively. The proportion of male patients in the treatment and control arms was 48.1%. Mean follow-up was 14.1 (SD 5.1) months for radiographic outcomes. Three trials reported the use of pulsed electromagnetic fields (PEMFs)^13,20,21^, 1 trial used capacitive coupling (CC)^19^, and 2 trials used direct current (DC)^15,16,36^. Furthermore, 1 trial had two intervention groups, of which one underwent PEMF and the other underwent DC^36^ (Table 1).

**Table 1:** Baseline characteristics of included trials.

Lead Author	Year	Country	Funding	Experimental Group				Control Group				Outcomes reported	Follow-up
				Mean age (years)	% Males	n	Lost/missing data	Mean age (years)	% Males	n	Lost/missing data		
Andersen	2000	Denmark	Corporate, Industry & Federal	68.9	38.1	44	6	71.5	31.0	33	4	Radiographic fusion rate, Dallas Pain Questionnaire, SF-36, Low Back Pain Rating Scale, walking distance	24 months
Foley	2008	U.S.A	None	46.9	55.2	122	41	46.7	53.1	118	42	Radiographic fusion rate, Mean visual analog scale, mean neck disability index, SF-12 physical health mean score	12 months
Goodwin	1999	U.S.A	Bioelectron Inc.	45	56.5	85	79	40.0	52.1	94	79	Radiographic & clinical fusion rate	12 months
Jenis	2000	U.S.A	N.R	53.0 (PEMF) 51.0 (DC)	50.0 (PEMF) 41.2 (DC)	22 (PEMF) 17 (DC)	0	47.1	63.6	22	0	Radiographic fusion grade, fusion mass bone density	12 months
Kane	1988	U.S.A	N.R	N.R	N.R	31	N.R	N.R	N.R	28	N.R	Radiographic fusion rate	18 months
Linovitz	2002	U.S.A	Corporate & Industry	56.77	40.8	97	21	56.6	36.4	104	21	Radiographic fusion rate	9 months
Mooney	1999	U.S.A	N.R	37.9	55.1	98	9	37.6	52.5	97	2	Radiographic fusion rate	12 months

Fusion success rate (FSR) was defined radiographically in all included trials^13,15,16,19–21,36,37^. The mean time in months for fusion assessment was 14.1 ± 5.1. FSR in smokers was reported in 5 trials^13,19–21,37^ and 4 trials included non-smokers^13,19,20,37^. FSR with respect to fusion level was reported in 4 trials for single level and for multiple fusion levels^13,20,21^.

There were 5 sham-controlled trials^13,15,16,19–21^, and 2 controlled trials^36,37^. The range of duration for treatment usage was a minimum of 3 months to a maximum of 9 months. Mean hours per day spent using the treatment, calculated by the authors, was 10 hours (Table 2).

**Table 2:** Details of electrical stimulation and control arms with odds ratio of fusion rate.

Lead Author	Date	Type of stimulation	Company name	Stimulator frequency (Hz), amplitude, other technical details	Treatment Frequency (hrs/day)	Treatment Duration	Treatment Fusion Rate (%)	Control details	Control Fusion Rate (%)	OR of Fusion Success Rate (Overall)	Change in Fusion Rate (Treatment – Control) (%)
Andersen	2009	DC	Biomet Spine SpF-XL 11b Spine Fusion Simulator	40 µA and 100 µA	24	6 months – 1 year after primary operation	64.3%	Dummy electrodes, identical	57.1%	1.35 (0.56, 3.25)	7.2%
Foley	2008	PEMF	Cervical-Stim® Osteogenesis Stimulator	N.R	4	3 months	83.6%	Inactive sham device	68.6%	2.33 (1.26, 4.32)	15%
Goodwin	1999	CC	SpinalPak from Biolectron, Inc.	60 kHz delivered via hydrogel surface electrodes	24	9 months	84.7%	Inactive sham device	64.9%	3.00 (1.45, 6.20)	20%
Jenis	2000	PEMF DC	PEMF – SpinalStim model 8212 DC - SpF2T stimulator	PEMF - Coil leads placed superficially over fusion site DC - N.R	PEMF – 2 DC - N.R	PEMF - 5 months DC - 5 months	97.4%	Control	95.5%	1.81 (0.11, 30.44)	1.9%
Kane	1988	DC	Osteostim HS11	5 µA at each of the four electrodes	N.R	22 weeks	80.6%	No implanted stimulator	53.6%	3.61 (1.13, 11.52)	27%
Linovitz	2002	PEMF	SpinaLogic, OrthoLogic, Tempe, AZ	Single coil worn posteriorly over fusion site	0.5	9 months	64.4%	Inactive sham device	43.3%	2.37 (1.34, 4.18)	21%
Mooney	1999	PEMF	Custom design stimulator (based on testing on rabbits)	Brace with multiple coils, 1.5 Hz, 1.8 G magnetic field	8	Until healed (although not specifically reported)	92.2%	Inactive sham device	67.9%	5.57 (1.89, 16.41)	24%

### Risk of bias

**Figure 2:**
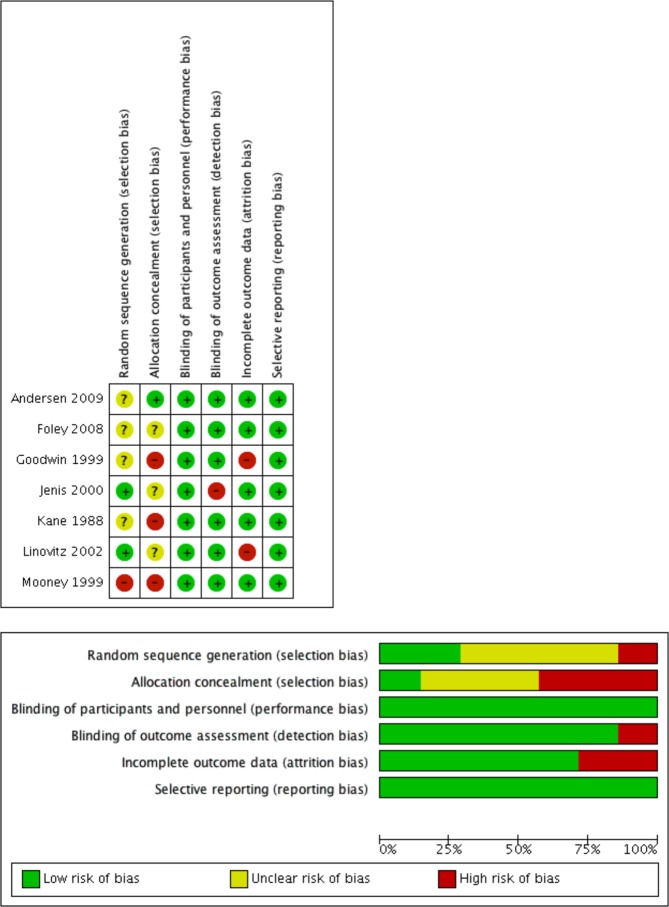
PRISMA Search Diagram.

The agreement for risk of bias assessment was high (*r* = 0.848, 95% CI: [0.716, 0.918], p < 0.0001). The risk of bias assessment (Table 2) is presented in Fig. 2. Publication bias was not significant (Egger’s test, p = 0.692; Begg’s test, p = 0.453).

### Effects of electrical stimulation on fusion rates

#### Fusion rates with stimulation overall

**Figure 3:**
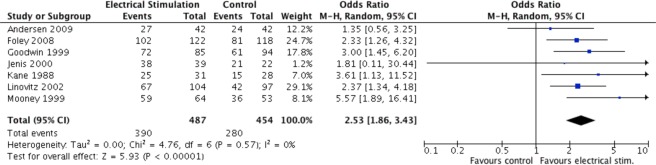
Pooled fusion success (OR) of electrical stimulation compared to no stimulation.

Electrical stimulation (PEMF, DC or CC) increased the odds of a successful fusion by 2.5 times relative to control (OR = 2.53, 95% CI 1.86 to 3.43, p < 0.00001), (Fig. 3).

#### fusion rates relative to smoking status

**Figure 4:**
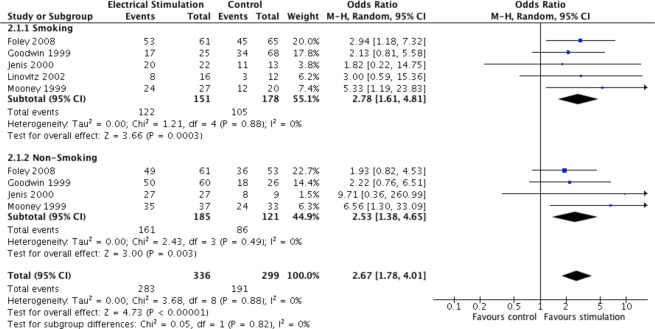
Pooled fusion success (OR) of electrical stimulation for smokers or non-smokers relative to no stimulation.

The odds of a successful fusion in smokers who received any of the three electrical stimulation methods were 2.8 times compared to smokers that received no stimulation (OR = 2.78, 95% CI 1.61 to 4.81, p = 0.0003 (Fig. 4). The odds of a successful fusion for non-smokers receiving electrical stimulation were 2.5 times the odds relative to non-smokers that received no electrical stimulation (OR = 2.53, 95% CI 1.38 to 4.65, p = 0.003).

#### Fusion rates relative to number of levels fused

**Figure 5:**
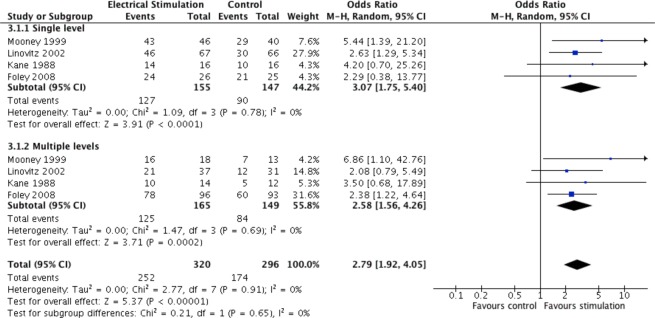
Pooled fusion success (OR) of electrical stimulation for single fusion or multi-fusion relative to no stimulation.

The odds of a successful single level fusion were 3.0 times greater in patients who received electrical stimulation compared to patients receiving no electrical stimulation (OR = 3.07, 95% CI 1.75 to 5.40, p < 0.0001), (Fig. 5). The odds of successful multi-level fusions were 2.6 times greater in patients receiving electrical stimulation relative to no electrical stimulation (OR = 2.58, 95% CI 1.56 to 4.26, p = 0.0002).

#### Fusion rates relative to stimulation method

**Figure 6:**
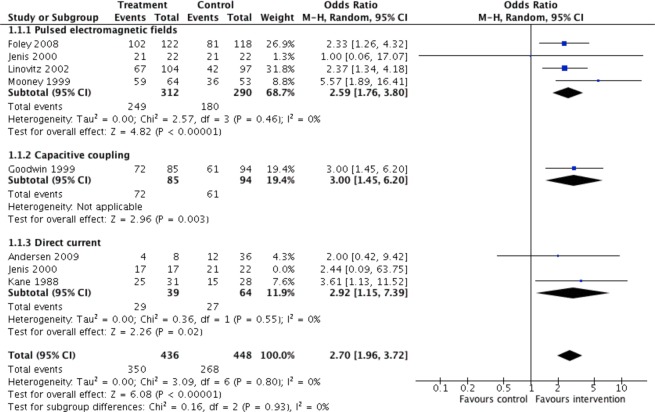
Pooled fusion success (OR) of electrical stimulation for type of stimulation relative to no stimulation. Jenis (2000) is a three arm trial including one PEMF arm, one DC arm, and one control arm. DC arm was excluded, to prevent duplicate counting of control group.

**Figure 7:**
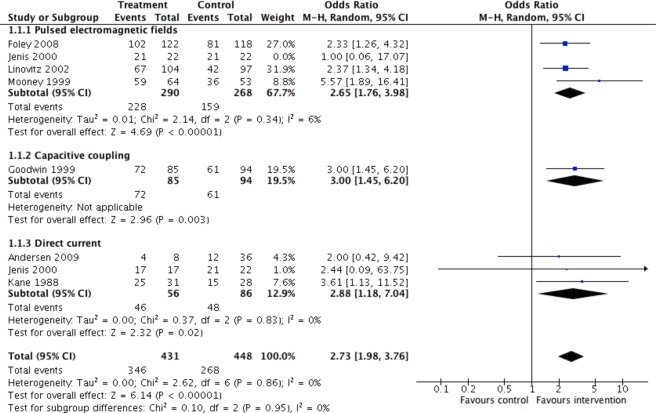
Pooled fusion success (OR) of electrical stimulation for type of stimulation relative to no stimulation. Jenis (2000) is a three arm trial including one PEMF arm, one DC arm, and one control arm. PEMF arm was excluded, to prevent duplicate counting of control group.

Capacitive coupling had the greatest odds for successful fusion relative to control (OR = 3.00, p = 0.003), followed by direct current (OR = 2.88, 95% CI 1.18 to 7.04, p = 0.02), and pulsed electromagnetic fields (OR = 2.59, 95% CI 1.76 to 3.80, p < 0.00001). As Jenis *et al*. is a three arm trial including one PEMF arm, one DC arm, and one control arm; the DC arm was excluded to prevent duplicate counting of control group (Fig. 6). Another forest plot was made that excluded the Jenis *et al*. PEMF arm to prevent duplicate counting of the control group (Fig. 7).

### Heterogeneity

There was negligible heterogeneity for the general comparison of stimulation to placebo for fusion success (I^2^ = 0.00%, p = 0.57). A similar trend for non-statistically significant heterogeneity was seen for fusion success comparisons of stimulation relative to placebo with respect to smoking (smoking - I^2^ = 0.00%, p = 0.80; non-smoking - I^2^ = 0.00%, p = 0.49), with respect to fusion level (single level - I^2^ = 0.00%, p = 0.78; multiple levels - I^2^ = 0.00%, p = 0.69), and with respect to stimulation type (PEMF - I^2^ = 0.00%, p = 0.46; DC - I^2^ = 0.00%, p = 0.83). Heterogeneity was not applicable for CC as there was only one trial within that subgroup.

### Sensitivity analysis

A sensitivity analysis was conducted to explore the impact of incomplete outcome data (Table 3). The analysis was based on the exclusion of Goodwin *et al*. and Linovitz *et al*.^19,21^, which were excluded on the grounds of high risk of bias in incomplete outcome data (Table 2). All percentage changes stated herein are relative to the corresponding aforementioned ORs for each analysis. The odds of successful fusion with PEMF stimulation relative to no stimulation increased by 10% (OR = 2.89, 95% CI 1.50 to 5.56, p = 0.001). The odds of a successful fusion in smokers receiving electrical stimulation relative to no electrical stimulation increased by 13% (OR = 3.20, 95% CI 1.54 to 6.63, p = 0.002). For the parallel comparison with non-smokers receiving electrical stimulation compared to those not receiving electrical stimulation, the increase was by 15% (OR = 2.97, 95% CI 1.55 to 5.00, p = 0.0006). The odds of a single successful fusion through electrical stimulation relative to no electrical stimulation increased by 24% (OR = 4.03, 95% CI 1.59 to 10.18, p = 0.003). For the parallel comparison examining multiple successful fusions in patients receiving electrical stimulation compared to those not receiving electrical stimulation, the odds increased by 7.5% (OR = 2.79, 95% CI 1.55 to 5.00, p = 0.0006). The overall effect of electrical stimulation compared to no electrical stimulation decreased by 10% (OR = 2.50, 95% CI 1.57 to 3.98, p = 0.0001).

**Table 3:** Results of the first sensitivity analysis on the basis of incomplete outcome data.

Analysis	OR (95% CI), p-value
***Stimulation type***	
PEMF stimulation	OR = 2.89 (1.50, 5.56), p = 0.001
***Smoking status***	
Smokers	OR = 3.20 (1.54, 6.63), p = 0.002
Non-smokers	OR = 2.97 (1.55, 5.00), p = 0.0006
***Fusion level***	
Single	OR = 4.03 (1.59, 10.18), p = 0.003
Multiple	OR = 2.79 (1.55, 5.00), p = 0.0006
***Overall effect***	
Overall	OR = 2.50 (1.57, 3.98), p = 0.0001

We conducted a second sensitivity analysis considering the variability in evaluating fusion (Table 4). We only included trials where outcome assessment was done by an independent blinded radiologist. This analysis was based on the exclusion of Andersen *et al*. and Jenis *et al*.^15,16,37^, which were excluded on the grounds of high risk of bias in incomplete outcome assessment (Table 2). The odds of successful fusion with DC stimulation relative to no stimulation increased by 20% (OR = 3.61, 95% CI 1.13 to 11.5, p = 0.03). The odds of a successful fusion in smokers receiving electrical stimulation relative to no electrical stimulation increased by 3.1% (OR = 2.87, 95% CI 1.63 to 5.07, p = 0.0003). For the parallel comparison with non-smokers receiving electrical stimulation compared to those not receiving electrical stimulation, a decrease by 4.3% was found (OR = 2.42, 95% CI 1.30 to 4.49, p = 0.005). Andersen *et al*. and Jenis *et al*.^15,16,37^ did not report fusion rate with respect to fusion level, and thus no sensitivity analysis was done for this. Finally, the overall effect of electrical stimulation compared to no electrical stimulation decreased by 0.72% (OR = 2.77, 95% CI 1.99 to 3.85, p < 0.00001).

**Table 4:** Results of the second sensitivity analysis on the basis of outcome assessment.

Analysis	OR (95% CI), p-value
***Stimulation type***	
DC stimulation	OR = 3.61 (1.13, 11.5), p = 0.03
***Smoking status***	
Smokers	OR = 2.87 (1.63, 5.07), p = 0.0003
Non-smokers	OR = 2.42 (1.30, 4.49), p = 0.005
***Overall effect***	
Overall	OR = 2.77 (1.99, 3.85), p < 0.00001

A third sensitivity analysis was conducted that limited the studies of inclusion to those with at least one year of follow-up (Table 5). Consequently, the study by Linovitz *et al*^[.21^, which had a follow-up of 9 months was excluded as per our initial inclusion criterion. The odds of successful fusion with PEMF stimulation compared to no stimulation increased by 12% (OR = 2.89, 95% CI 1.50 to 5.56, p = 0.001). The odds of a successful fusion in smokers receiving electrical stimulation relative to no electrical stimulation decreased by 0.72% (OR = 2.76, 95% CI 1.54 to 4.93, p = 0.0006). Linovitz *et al*.^21^ did not report pain severity, and thus no sensitivity analysis was done for this. The odds of a successful fusion at a single level with electrical stimulation compared to no electrical stimulation increased by 31% (OR = 4.03, 95% CI 1.59 to 10.18, p = 0.003). For the parallel comparison of multiple fusion levels with electrical stimulation compared to without, the odds of a successful fusion increased by 8.1% (OR = 2.79, 95% CI 1.55 to 5.00, p = 0.0006). Finally, the overall effect of electrical stimulation compared to no electrical stimulation increased by 2.4% (OR = 2.59, 95% CI 1.80 to 3.73, p < 0.00001).

**Table 5:** Results of the second sensitivity analysis on the basis of outcome assessment.

Analysis	OR (95% CI), p-value
***Stimulation type***	
1.1.1 PEMF stimulation	OR = 2.89 (1.50, 5.56), p = 0.001
***Smoking status***	
2.1.1 Smoking	OR = 2.76 (1.54, 4.93), p = 0.0006
***Fusion level***	
3.1.1 Single level	OR = 4.03 (1.59, 10.18), p = 0.003
3.1.2 Multiple levels	OR = 2.79 (1.55, 5.00), p = 0.0006
***Overall effect***	
Overall	OR = 2.59 (1.80, 3.73), p < 0.00001

### Subgroup differences

Our subgroup analysis results for type of stimulation were as follows: Capacitive coupling had the greatest odds for successful fusion relative to control (OR = 3.00, p = 0.003), followed by direct current (OR = 2.88, 95% CI 1.18 to 7.04, p = 0.02), and pulsed electromagnetic fields (OR = 2.59, 95% CI 1.76 to 3.80, p < 0.00001). However, the test for subgroup interaction by stimulation type, smoking status, and number of levels fused were all non-significant (p = 0.93). The assumption of varying efficacy based on stimulation type was tested, and no significant differences were noted.

### Assessment of the evidence (GRADE)

The outcome of fusion success rate was rated as moderate quality evidence due to indirectness (Table 8). There was a high directness in population and intervention, notable indirectness with outcomes, and little indirectness with follow-up. Outcomes of pain and function were only reported in 2 trials, precluding meta-analysis. All but one study had a follow-up of at least 12 months or more. Hence, with some limitations, the studies directly address the review question. A summary of our findings can be found in Table 9.

## Discussion

Our meta-analysis of randomized controlled trials found moderate quality evidence for electrical stimulation after spinal fusion surgery in improving radiographically defined fusion rates. Our hypothesis of observing a consistent trend of positive treatment effect from electrical stimulation was supported by our analyses.

Further, regardless of smoking status, fusion level (single or multiple), and the particular type of electrical stimulation, moderate quality evidence found that electrical stimulation in general leads to greater fusion success rates compared to no stimulation. Two previous reviews are consistent with our review^22,26^. Akai *et al*. conducted a review with the same outcome of radiographic fusion success rate, but included low quality observational, case-series, and case-control studies in addition to randomized trials. Akai *et al*.’s, much like Tian *et al*.'s, findings both show a significant effect with the use of electrical stimulation in spinal fusion surgery. Tian *et al*. conducted subgroup analyses to conclude, similarly to our review, that treatment effect on radiographic fusion success rate did not differ by smoking status or fusion levels. However, Tian *et al*.'s review is limited by an incomprehensive search and lack of quality assessment. The present results are also in keeping with our previous study assessing the efficacy of electrical stimulation for bone healing. That study however, was limited as the inclusion criteria were broad and included acute fractures, nonunions, osteotomies, and spinal fusions. When spinal fusion data was parsed out, rates of radiographic nonunion were found to be highly in favor of electrical stimulation (Mean Difference 0.62, CI 0.45–0.84).

We wish to highlight that our moderate quality evidence is supported by a highly statistically significant effect (p < 0.00001), a narrow confidence interval (1.86, 3.43) and the inclusion of only high quality evidence (randomized placebo or sham-controlled trials). Our review includes the largest number of trials to date evaluating fusion success rates with postoperative electrical stimulation after spine fusion surgery. Our robust findings build on the weaker findings of previous reviews, and present moderate quality of evidence demonstrating the notable efficacy of electrical stimulation for spinal fusion.

Although electrical stimulation has been found to be helpful in improving bone healing and fusion rates, some safety concerns are shared amongst treating orthopaedic surgeons^38,39^. Clinicians are primarily concerned with electrode migration, infection, and electrode failure when it comes to internal electrical stimulation, including capacitive coupling and direct current electrical stimulation^38^. Suspected when there is a change in the area of induced paresthesia, electrode migration is the most common complication^38^. In many instances, this problem may be resolved by adjusting the stimulator parameters or reprogramming of the stimulator^38^. With infection rates ranging from 2.5% to 14%, infection is the costliest ‘safety’ concern related to this therapeutic measure^38^. The risk of infection may be reduced if care is taken to create a sterile surgical environment, minimizing hospital stay, administration of antimicrobial prophylaxis, limiting exposure of the wound and ensuring proper wound care^38^. As a result, while there are some concerns regarding electrical stimulation’s safety, these are minimal and may be minimized with proactive care^38,39^.

### Limitations

We conducted multiple subgroup analyses to determine if any large differences or similarities in the magnitude of treatment effect exist between patients with differing characteristics^28,40^. Although these subgroup analyses were pre-specified, this is also the main methodological limitation of our review as there is a theoretically increased chance of false negative and/or false positive findings with an increasing number of subgroup analyses^28^. The Cochrane collaboration recommends conducting subgroup analyses when at least 10 studies are included in the meta-analysis. Although this number is arbitrary, we recognize that conducting subgroup analyses with a lesser number of studies may not be appropriate. However, we opted to conduct subgroup analyses provided the paucity of evidence, as only 7 studies were included precluding meta-analysing any other outcome aside from radiographic fusion. We report our subgroup findings in the results section, but it is important to note that the tests for interaction were all non-statistically significant, indicating there is no difference in the positive treatment effect with respect to type of stimulation (p = 0.93), smoking status (p = 0.82), fusion level (p = 0.65). The modifying effect of age on the treatment effect is another interesting subgroup category, although it was not specifically tested for considering the aforementioned limitations. Considering the possible interaction between age and treatment effect on fusion rates, further studies are alerted to consider this potential subgroup.

Additionally, a primary focus of radiographically measured outcomes, such as non-union, is a shared limitation with previous reviews^22,26,41^. Although we set out to determine the effect of electrical stimulation on patient-important outcomes as well, we found a paucity of evidence focusing on pain and function. Of the 7 included studies, pain was reported in only 1 trial^15,16^, and as an adverse event in another trial^20^. This highlights a definite gap in the literature and area for further study.

### Implications for clinical practice and research

Previous reviews report the inability to extend their findings to clinical practice due to poor methods and low quality evidence^22,26^. Our results are supported by a notably high statistically significant effect, a narrow confidence interval, and the inclusion of only high quality randomized trials with human subjects. This review supports the utility of postoperative electrical stimulation as an adjunctive therapy for improving radiographically defined fusion success rates through moderate quality evidence. This may be particularly beneficial in patients that present fusion challenges, such as smokers or multi-level fusions, although further research into subgroup effects is required. Although this review has important implications for clinical practice relating to the outcome of fusion success, further research through high-quality randomized trials is needed to establish the efficacy of electrical stimulation on pain and functional outcomes^42^.

## Conclusion

This systematic review and meta-analysis found moderate-level evidence supporting the use of postoperative electrical stimulation as an adjunct to spinal fusion surgery. When compared to sham, placebo-controlled, or no stimulation, patients treated with postoperative electrical stimulation have significantly greater rates of successful radiographically defined fusions.

## Supplementary information

Supplementary information
