# Supplementary material for: Efficacy of Electrical Stimulation for Spinal Fusion: A Systematic Review and Meta-Analysis of Randomized Controlled Trials
Source: Sci Rep. 2020 Mar 12;10:4568. doi: 10.1038/s41598-020-61266-x (PMC7067864; doi:10.1038/s41598-020-61266-x)
Supplement: Supplementary file 1 — Supplementary information [file 41598_2020_61266_MOESM1_ESM.docx]

**Efficacy of Electrical Stimulation for Spinal Fusion: A Systematic Review and Meta-Analysis of Randomized Controlled Trials**

Shakib Akhter BScN, MSc^1,2*^, Abdul Rehman Qureshi HBSc^1^, Idris Aleem MEng, Hussein Ali El-Khechen HBSc, Shadman Khan BHSc(c), Omaike Sikder BSc(c), Moin Khan MD, MSc^2^, Mohit Bhandari MD, PhD^1,2,3^, Ilyas Aleem MD, MSc^4^

**Investigation preformed at**: Department of Health Research Methods, Evidence, and Impact, McMaster University

^1^Department of Health Research Methods, Evidence, and Impact, McMaster University

^2^Department of Orthopaedic Surgery, McMaster University

^3^OrthoEvidence, Burlington, Ontario

^4^Department of Orthopaedic Surgery, University of Michigan

*Corresponding Author:

Shakib Akhter

 Department of Health Research Methods, Evidence, and Impact, McMaster University

1280 Main Street West, Hamilton, ON L8S 4K1, Canada

[shakib.akhter@gmail.com](mailto:shakib.akhter@gmail.com)

+1 (647) 760- 2026

**Appendices**

**Figure 1:** PRISMA Search Diagram

**
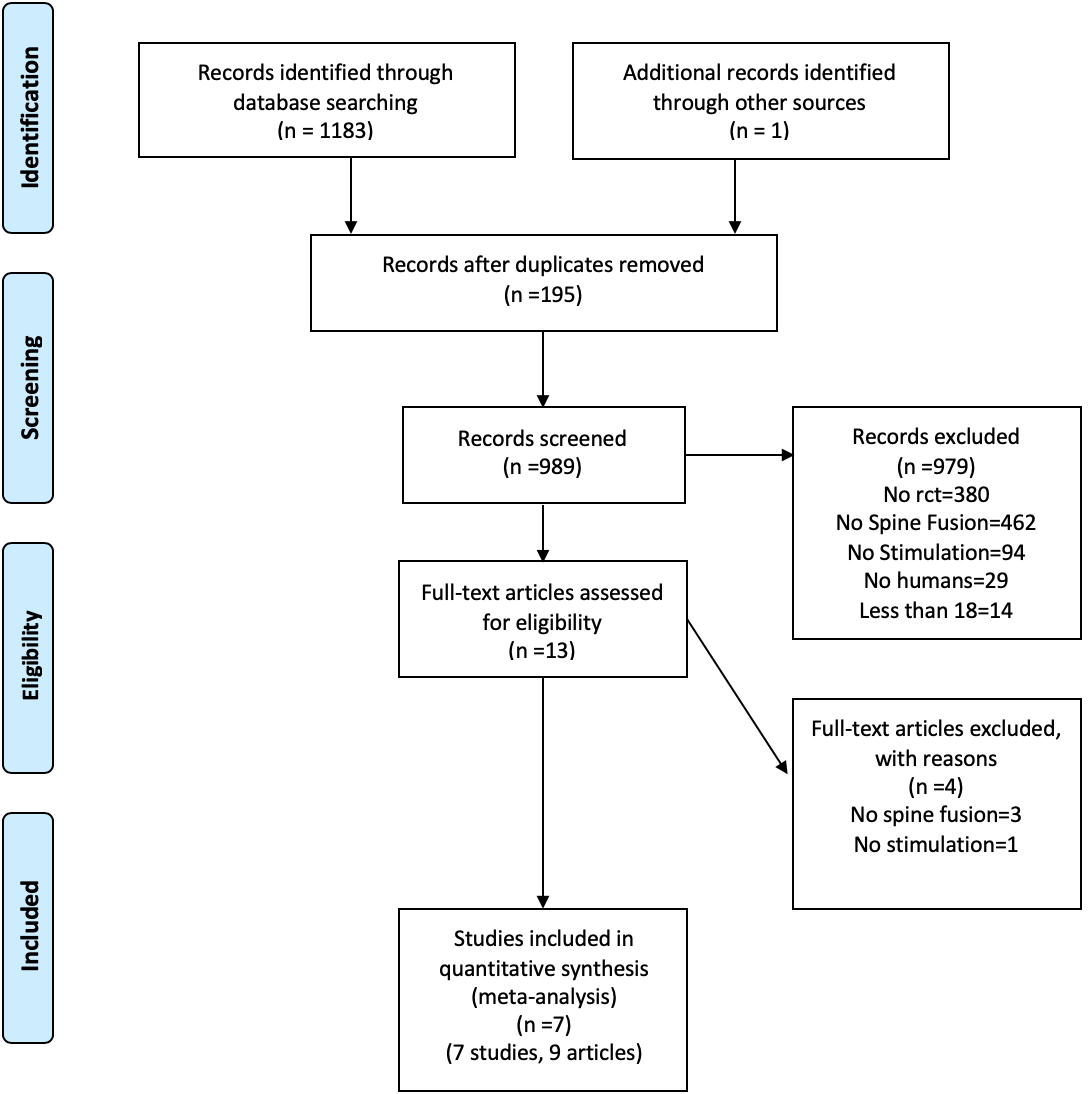
**

**Figure 2.** Risk of bias assessment

**
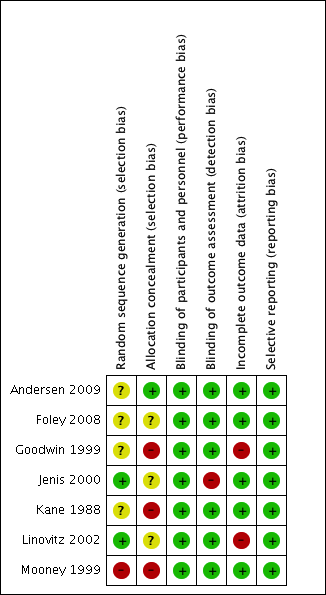
**

**
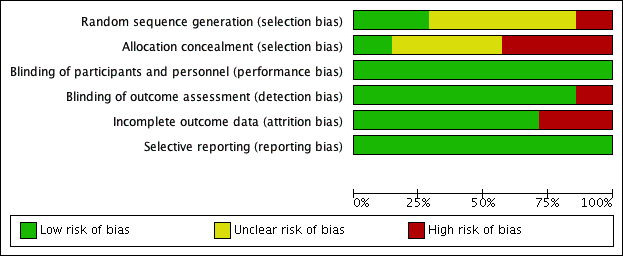
**

**Figure 3.** Pooled fusion success (OR) of electrical stimulation compared to no stimulation.

**
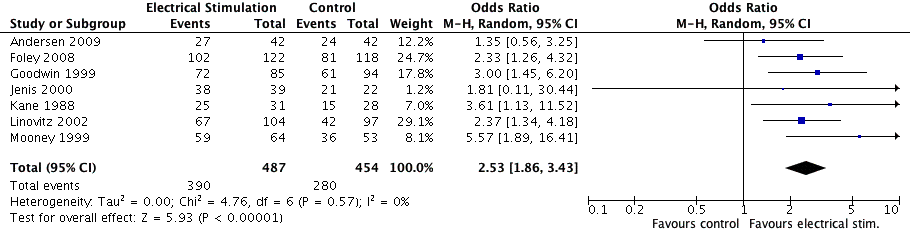
**

**Figure 4.** Pooled fusion success (OR) of electrical stimulation for smokers or non-smokers relative to no stimulation.

**
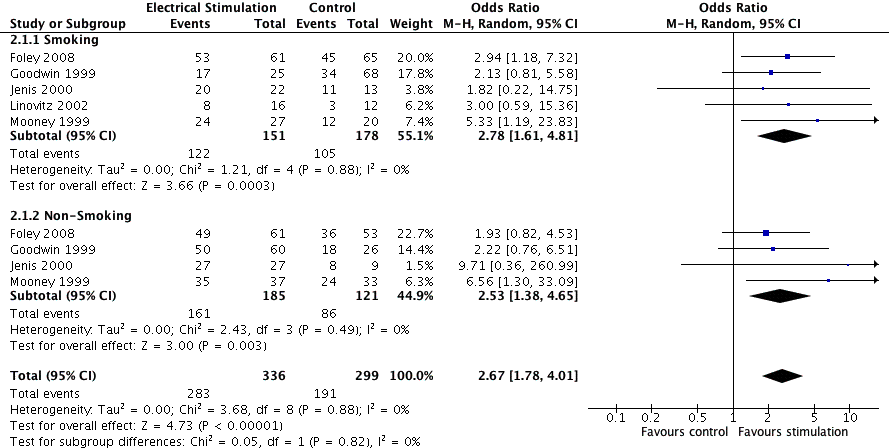
**

**Figure 5.** Pooled fusion success (OR) of electrical stimulation for single fusion or multi-fusion relative to no stimulation.

**
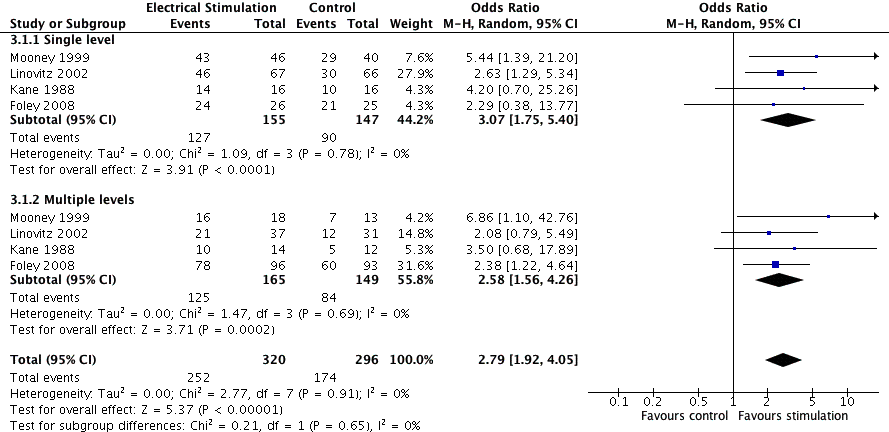
**

**Figure 6.** Pooled fusion success (OR) of electrical stimulation for type of stimulation relative to no stimulation. Jenis (2000) is a three arm trial including one PEMF arm, one DC arm, and one control arm. DC arm was excluded, to prevent duplicate counting of control group.

**
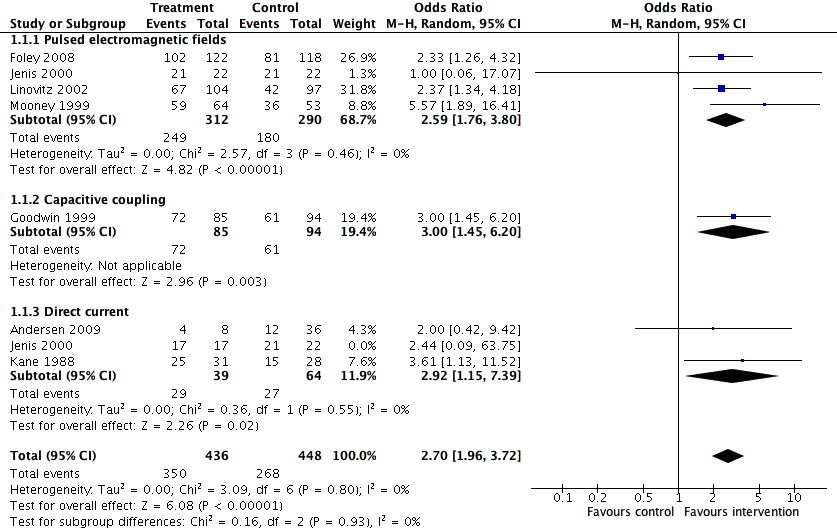
**

**Figure 7.** Pooled fusion success (OR) of electrical stimulation for type of stimulation relative to no stimulation. Jenis (2000) is a three arm trial including one PEMF arm, one DC arm, and one control arm. PEMF arm was excluded, to prevent duplicate counting of control group.

**
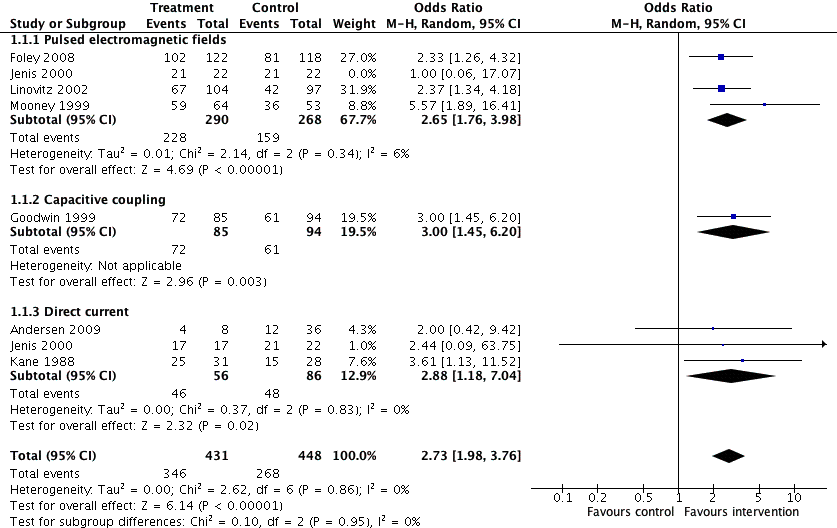
**

**Table 1: Search strategies**

| CINAHL | CENTRAL | **EMBASE** | **MEDLINE** |
| --- | --- | --- | --- |
| S12. S9 AND S10 AND S11  S11. S4 OR S5 OR S6 OR S7 ORS8  S10. S1 OR S2 OR S3  S9. (MH "Randomized Controlled Trials") OR (MH "Clinical Trials+")  S8. (MH "Orthooedics") OR (MH "Orthopedic Surgery+")  S7. (MH "Spine+")  S6. (MH "Fractures, Ununited+")OR "nonunion"  s5. (MH "Spinal Fractures")  S4. (MH "Spinal Fusion") OR spinal fusion  S3. (MH "Magnet Therapy") OR (MH "Magnet Fields+")OR "magnet field therapy"  S2. "direct current"  S1. (MH "Electric Stimulation") OR "electrical stimulation or estim" | #1 MeSH descriptor: [Electric Stimulation] explode all trees  #2 Electromagnetic fields  #4 Direct current  #5 Capacitive coupling  #6 Inductive coupling  #7 Magnetic field therapy  #8 Spinal fusion  #9 Spine surgery  #10 Fracture  #11 Nonunion  #12 Delayed union  #13 Fracture healing  #14 #1 or #2 or #3 or #4 or #5 or  #6 or #7  #15 #8 or #9 or #10 or #11 or  #12 or #13  #16 #14 and #15 | 1. exp electrostimulation/ 2. electric$ stimulation.mp.  3. exp functional electrical stimulation/  4. exp electrotherapy/  5. electrotherapy.mp.  6. exp low frequency electrotherapy/ or exp high frequency electrotherapy/  7. Capacitive coupling.mp.  8. Inductive coupling.mp.  9. Direct current.mp. or direct current/  10. electromagnetic fields.mp. or exp electromagnetism/  11. exp electromagnetic field/ or exp pulsed electric field/  12. exp magnetotherapy  13. spine surgery.mp. or exp spine surgery/  14. spin$ surgery.mp.  15. spin$ fusion.mp.  16. Cervical Vertebrae.mp. or exp cervical vertebra/  17. Thoracic vertebrae.mp. or exp thoracic vertebra/  18. Lumbar vertebrae.mp. or exp lumbar vertebra/  19. exp cervical spine/ or exp spine/ or exp thoracic spine/ or exp anterior spine fusion/ or exp lumbar spine/ or exp posterior spine fusion/  20. 1 or 2 or 3 or 4 or 5 or 6 or 7 or 8 or 9 or 10 or 11 or 12  21. 13 or 14 or 15 or 16 or 17 or 18 or 19  22. trial.ti,ab.  23. abstract.ti,ab.  24. random$.ti,ab.  25. randomized controlled trial/  26. exp controlled clinical trial/  27. 22 or 23 or 24 or 25 or 26  28. 20 and 21 and 27  29. limit 28 to human | 1. electric$ stimulation.mp.  2. exp Electric Stimulation/  3. electrical stimulation therapy.mp.  4. exp Electric Stimulation Therapy/  5. electromagnetic field$.mp. [  6. exp Electromagnetic Fields/  7. Capacitive coupling.mp.  8. Direct current.mp.  9. Inductive coupling.mp.  10. Pulsed electromagnetic field$.mp.  11. electric$ stimulat$.mp.  12. Magnetic Field Therapy/  13. Magnetic field therapy.mp.  14. 1 or 2 or 3 or 4 or 5 or 6 or 7 or 8 or 9 or 10 or 11 or 12 or 13  15. spin$ surgery.mp.  16. spin$ fusion.mp.  17. Cervical Vertebrae/  18. Thoracic Vertebrae/  19. Lumbar Vertebrae/  20. Spine.mp.  21. exp SPINE/  22. exp "Bone and Bones"/  23. exp ORTHOPEDICS/  24. 15 or 16 or 17 or 18 or 19 or 20 or 21 or 22 or 23  25. randomized controlled trial.mp.  26. controlled clinical trial.mp.  27. randomized.mp.  28. placebo.mp.  29. drug therapy.mp.  30. randomly.mp.  31. trial.mp.  32. groups.mp.  33. 25 or 26 or 27 or 28 or 29 or 30 or 31 or 32  34. exp animals/ not humans.sh.  35. 33 not 34  36. 14 and 24 and 35 |

**Table 2:** Detailed risk of bias assessment

| **Bias domain** | **Authors Judgment** | **Support for Judgment** | |
| --- | --- | --- | --- |
| **Anderson (2009)** | | | |
| Random sequence generation (selection bias) | Unclear | Direct evidence | “patients were randomized to posterolateral spinal fusion, using fresh frozen allograft, with or without 40 A DC-electrical stimulation in a 1:1 fashion… a new device was incorporated in the study as a third arm with a 1:1:2 randomization among control, 40A and 100A.” |
|  |  | Rationale | There is no mention of how authors generated the random sequence through any means in either paper. We cannot conclude whether it is low or high risk due to insufficient information, therefore we judge it as unclear. |
| Allocation concealment (selection bias) | Low risk | Direct evidence | “Randomization was performed before surgery in the out-patient clinic at each center by means of sealed envelopes after the patient’s informed consent had been obtained.” |
|  |  | Rationale | Although there was no mention if envelopes were opaque and/or sequentially numbered, there was concealment through a sealed envelope |
| Blinding of participants and personnel (performance bias)  ~fusion rates | Low Risk | Direct evidence | “…dummy electrodes were used in the control group. They were completely identical to those provided with the stimulator device and were provided by the manufacturer of the stimulator”  ^“^…neither the patient nor the surgeon was blinded to treatment group” |
|  |  | Rationale | Participants in the control group were blinded. Participants in the treatment group were not blinded, which may lead to performance bias. However, given the nature of the intervention, it is unlikely that patient/physician performance will alter the outcome, so we determine this to be low risk. |
| Blinding of outcome assessment (detection bias)  ~ fusion rates | Low Risk | Direct evidence | “Review of the scans was blinded to treatment group” |
|  |  | Rationale | Outcome assessors were completely blinded to intervention received. As per the Cochrane handbook, blinded outcome assessment is a low risk of bias. |
| Incomplete outcome data (attrition bias)  ~ fusion rates | Low risk | Direct evidence | “includes 95 patients who underwent at least 1-year radiologic follow-up; baseline characteristics are seen in Table 1. One patient in the control group died after 1-year control, leaving 94 patients available for the 2-year follow-up CT scan. Of these patients, 84 (89%) underwent CT scan at 2-year follow-up. Excluded patients are described in Table 2.” |
|  |  | Rationale | For the fusion rates outcome, authors reported attrition and exclusion rates. Specific details regarding age, sex, which treatment group, diagnosis, and cause of dropout were provided in Table 2. Appropriate reporting and handling of incomplete outcome data and a low attrition rate (balanced between groups) of 11% are reflective of a low risk of bias. |
| Selective reporting (reporting bias) | Low risk | Direct evidence | *Results^2^* section:  “Very low fusion rates based on the high resolution CT were observed (Table 3).”  “Fusion rates revealed by plain radiographs were 57% (24/42) in the control group, 64% (27/42) in the 40 μA group and 36% (4/11) in the 100 μA group (not significant).”^2^ |
|  |  | Rationale | Authors reported all information pertinent to the outcome in strenuous detail. Table 3 reports each of the 84 patients fusion rates and classification of non-unions according to their study group. Table 4 further explains fusion status with respect to known risk factors (gender, age, etc) for each patient. No evidence of selective reporting, therefore low risk of bias. |
| **Foley (2008)** | | | |
| Random sequence generation (selection bias) | Unclear | Direct evidence | “were randomly assigned to one of  two surgical groups: those who received PEMF stimulation and those who did not.” |
|  |  | Rationale | There is no mention of how authors generated the random sequence through any means in either paper. We cannot conclude whether it is low or high risk due to insufficient information, therefore we judge it as unclear. |
| Allocation concealment (selection bias) | Unclear | Direct evidence | “were randomly assigned to one of  two surgical groups: those who received PEMF stimulation and those who did not.” |
|  |  | Rationale | There is no mention of how authors concealed allocation through any means in either paper. We cannot conclude whether it is low or high risk due to insufficient information, therefore we judge it as unclear. |
| Blinding of participants and personnel (performance bias)  ~fusion rates | Low Risk | Direct evidence | Patients received either “PEMF stimulation or no stimulation after surgery” |
|  |  | Rationale | Neither participants or personnel were blinded. However, given the nature of the intervention, it is unlikely that patient/physician performance will alter the outcome, so we determine this to be low risk. |
| Blinding of outcome assessment (detection bias)  ~ fusion rates | Low Risk | Direct evidence | “Two orthopedic surgeons not otherwise affiliated  with the study and blinded to treatment group evaluated the radiographs, as did a blinded radiolo-  gist.” |
|  |  | Rationale | Outcome assessors were completely blinded to intervention received. As per the Cochrane handbook, blinded outcome assessment is a low risk of bias. |
| Incomplete outcome data (attrition bias)  ~ fusion rates | Low Risk | Direct evidence | “In the control group, 118 (73.8%) patients were evaluable at 6 months postoperatively. Similarly, 122 (74.9%) patients in the PEMF group were evaluable at that time point.”  “An intent-to-treat analysis was performed  to compare the actual results of the study with those obtained when various assumptions are made regarding the outcomes of patients who did not complete the study (Table 3)” |
|  |  | Rationale | Authors reported balanced loss to follow-up rates. Specific details about missing patients and fusion rates are provided to Table 3. Appropriate reporting and handling of incomplete outcome data and attrition rate (balanced between groups) are reflective of a low risk of bias. |
| Selective reporting (reporting bias) | Low Risk | Direct evidence | “At 6 months after surgery, 81/118 (68.6%) patients in the control group had fused, as judged by the study criteria described, and 102/122 (83.6%) patients in the PEMF group had fused (p5.0065). For the patients available for follow-up at 12 months, radiographically confirmed fusion was achieved in 104/120 (86.7%) of the control patients and 116/125 (92.8%) of the PEMF patients (p5.1129).” |
|  |  | Rationale | Authors reported all information pertinent to the outcome in strenuous detail. Table 4 reports outcome details with respect to clinical and demographic characteristics. Table 5 further explores fusion status with respect to demographic characteristics. There is no evidence of selective reporting, therefore low risk of bias. |
| **Goodwin (1999)** | | | |
| Random sequence generation (selection bias) | Unclear | Direct evidence | “The patients were randomized within 3 weeks of the fusion surgery” |
|  |  | Rationale | There is no mention of how authors generated the random sequence through any means in either paper. We cannot conclude whether it is low or high risk due to insufficient information, therefore we judge it as unclear. |
| Allocation concealment (selection bias) | High risk | Direct evidence | “The patients were randomized within 3 weeks of the fusion surgery” |
|  |  | Rationale | There is no mention of how authors concealed allocation through any means in either paper. |
| Blinding of participants and personnel (performance bias)  ~fusion rates | Low Risk | Direct evidence | “compare active to placebo stimulators” |
|  |  | Rationale | Blinding of participants and personnel via Sham stimulation |
| Blinding of outcome assessment (detection bias)  ~ fusion rates | Low Risk | Direct evidence | Outcomes assessed by “Investigator and blinded independent radiologist” |
|  |  | Rationale | Outcome assessors were completely blinded to intervention received. As per the Cochrane handbook, blinded outcome assessment is a low risk of bias. |
| Incomplete outcome data (attrition bias)  ~ fusion rates | High Risk | Direct evidence | “24 investigators enrolled 337 patients…179 completed independent radiographic review” |
|  |  | Rationale | 47% imbalanced loss to follow-up between groups and no intention to treat analysis. This creates possibility for significant bias, this high risk. |
| Selective reporting (reporting bias) | Low Risk | Direct evidence | “Table 4 lists active and success rates for the active and placebo group, with stratification…Table 6 describes the internal fixation strata further subdivided to show the coexisting impact of multilevel fusions and smoking on physician-reported success rates” |
|  |  | Rationale | Authors reported all information pertinent to the outcome in strenuous detail. Table 5 reports all outcome data stratified by type of procedure, smoking, status, and fusion level for both groups. Table 6 further explores smoking and fusion level subgroups by internal fixation procedure. |
| **Kane (1988)** | | | |
| Random sequence generation (selection bias) | Unclear | Direct evidence | “Randomly allocated to receive either a spinal fusion procedure without stimulation (control), or the same spinal fusion procedure with the addition of the direct current bone growth stimulator (treatment). Randomization was in blocks of 4” |
|  |  | Rationale | Although there is discussion of a randomization sequence, there is no discussion of how it was generated (i.e computer software). We cannot conclude whether it is low or high risk due to insufficient information, therefore we judge it as unclear. |
| Allocation concealment (selection bias) | High Risk | Direct evidence | “Randomly allocated to receive either a spinal fusion procedure without stimulation (control), or the same spinal fusion procedure with the addition of the direct current bone growth stimulator (treatment).” |
|  |  | Rationale | There is no mention of how authors concealed allocation through any means in either paper. Coupled with an unclear judgement of random sequence generation, we believe this is high risk. |
| Blinding of participants and personnel (performance bias)  ~fusion rates | Low Risk | Direct evidence | Radiographically successful spinal fusion was higher when the direct current implantable stimulator was used as an adjunct to the conventional spinal fusion procedure, then when surgery alone was performed |
|  |  | Rationale | Neither participants or personnel were blinded. However, given the nature of the intervention, it is unlikely that patient/physician performance will alter the outcome, so we determine this to be low risk. |
| Blinding of outcome assessment (detection bias)  ~ fusion rates | Low Risk | Direct evidence | “The radiographic assessment was confirmed by an independent radiologist to assure a lack of investigator bias” |
|  |  | Rationale | Outcome assessors were completely blinded to intervention received. As per the Cochrane handbook, blinded outcome assessment is a low risk of bias. |
| Incomplete outcome data (attrition bias)  ~ fusion rates | Low Risk | Direct evidence | Of the 63 patients, 59 patients were available for follow-up, from 7 investigators. There were 28 controlled patients and 31 stimulated patients. |
|  |  | Rationale | Authors reported very low and balanced loss to follow-up rates. Specific details on outcomes are provided. Appropriate reporting and handling of incomplete outcome data and attrition rate (balanced between groups) are reflective of a low risk of bias. |
| Selective reporting (reporting bias) | Low Risk | Direct evidence | “Tables 1, 2 and 3 indicate the patient characteristics for each group by age, and three criteria, and number of levels used” |
|  |  | Rationale | Authors reported all information pertinent to the outcome in strenuous detail. All data for all outcomes reported for all patients. |
| **Jenis (2000)** | | | |
| Random sequence generation (selection bias) | Low Risk | Direct evidence | “The randomization schedule was computer generated before the initiation of the study” |
|  |  | Rationale | Authors used a computer generated randomization sequence, this low risk of bias |
| Allocation concealment (selection bias) | Unclear | Direct evidence | “randomized to one of three treatment protocols: 1) adjunctive PEMF group (n = 22) fitted with Spinal-Stim model 8212(AME) within 30 days of surgery; 2) DC group (n = 17) had a SpF-2T stimulator(EBI) implanted at the time of surgery; or 3) control group (n = 22).” |
|  |  | Rationale | There is no mention of how authors concealed allocation through any means in either paper. We cannot conclude whether it is low or high risk due to insufficient information, therefore we judge it as unclear. |
| Blinding of participants and personnel (performance bias)  ~fusion rates | Low Risk | Direct evidence | “randomized to… control group (n = 22).” |
|  |  | Rationale | Neither participants or personnel were blinded. However, given the nature of the intervention, it is unlikely that patient/physician performance will alter the outcome, so we determine this to be low risk. |
| Blinding of outcome assessment (detection bias)  ~ fusion rates | High Risk | Direct evidence | “Masking of the analysis was impossible because of the presence of the electrical leads on the radiographs in the DC treatment group.” |
|  |  | Rationale | Outcome assessors were not blinded. An independent radiologist was not used to confirm results. Subjective clinical and radiographic judgment of fusion rates presents a high risk of bias. |
| Incomplete outcome data (attrition bias)  ~ fusion rates | Low Risk | Direct evidence | “Twenty-two patients were randomized into the nonstimulated treatment group… Twenty-two patients were randomized into the PEMF treatment Group... Seventeen patients were randomized into the DC electrical stimulation group.”  Outcome data: “Data for nonstimulated group (n=22)”, “Data for direct current group (n=17)”, “Data for pulsed electromagnetic field therapy (n=22)” |
|  |  | Rationale | There was no loss to follow-up. Authors reported all information pertinent to the outcome in strenuous detail. All data for all outcomes reported for all patients. |
| Selective reporting (reporting bias) | Low Risk | Direct evidence | “The non stimulated group was determined as follows: 4.7% grade 1, 14.3% grade 2, and 81% grade 3. The PEMF group was 5% grade 1, 30% grade 2, and 65% grade 3, respectively. Fusion rates for DC group was 0% grade 1, 38.9% grade 2, and 61.1% grade .” |
|  |  | Rationale | All outcome data was reported in detail and stratified by fusion level and smoking history. Tables 1-3 display all outcomes stratified as above for control, DC, and PEMF, respectively. |
| **Mooney (1990)** | | | |
| Random sequence generation (selection bias) | High Risk | Direct evidence | “A randomized double-blind prospective study of pulsed electromagnetic fields for lumbar interbody Fusion was performed on 195 subjects” |
|  |  | Rationale | Aside from above, there is no further discussion of randomization or sequence generation throughout the article. There is no mention of how authors generated the random sequence through any means. |
| Allocation concealment (selection bias) | High Risk | Direct evidence | “A randomized double-blind prospective study of pulsed electromagnetic fields for lumbar interbody Fusion was performed on 195 subjects” |
|  |  | Rationale | Aside from above, there is no further discussion of randomization, sequence generation, or allocation throughout the article. There is no mention of how authors concealed allocation through any means. |
| Blinding of participants and personnel (performance bias)  ~fusion rates | Low Risk | Direct evidence | ^“^After surgery, patients were fitted with a special brace with electromagnetic coils... Neither the patient nor the surgeon was aware of the function and characteristics of the brace. They were aware, however, that the brace itself may or may not be functioning” |
|  |  | Rationale | This was a double blind trial where patients and physicians were blinded to intervention and sham control. |
| Blinding of outcome assessment (detection bias)  ~ fusion rates | Low Risk | Direct evidence | “The surgeon identified the radiographic status at the time of union, but an independent blinded radiologist confirmed this reading” |
|  |  | Rationale | There was an element of blinding outcome assessors. |
| Incomplete outcome data (attrition bias)  ~ fusion rates | Low Risk | Direct evidence | “A total of 195 patients completed the study. There were 98 patients in the active group and 97 patient in the control group… “Originally they were 107 patient to the active control group in 99 patients in the placebo group…” |
|  |  | Rationale | Most patients completed the study and there were very few losses to follow-up. |
| Selective reporting (reporting bias) | Low Risk | Direct evidence | In those 64 patients randomized to the active group who consistently use the device, there was a 92.2% success rate. In those patients who are inconsistent (less than 4 hours use a day) in the use of the equipment, there was a similar success rate to the placebo group, which had an overall success rate of 64.9%” |
|  |  | Rationale | All outcomes for all patients where reported in strenuous detail. |
| **Linovitz (2002)** | | | |
| Random sequence generation (selection bias) | Low Risk | Direct evidence | Randomization was accomplished by a computer-generated randomization code provided by an independent third party. A six-block randomization code was used to eliminate bias between investigational sites based on enrollment rates. |
|  |  | Rationale | Authors used a computer to generate their randomization sequence. |
| Allocation concealment (selection bias) | Unclear | Direct evidence | There is no discussion of how allocation was concealed. We cannot conclude whether it is low or high risk due to insufficient information, therefore we judge it as unclear. |
|  |  | Rationale | Authors included no direct discussion of allocation concealment. However, they reported the details of sham stimulation, which indicate that allocation |
| Blinding of participants and personnel (performance bias)  ~fusion rates | Low Risk | Direct evidence | “All treating physicians, reviewers, patients, and the sponsor were blinded as to the activity status of all devices.” |
|  |  | Rationale | Blinding of participants and personnel via Sham stimulation |
| Blinding of outcome assessment (detection bias)  ~ fusion rates | Low Risk | Direct evidence | “The evaluation of fusion outcome was performed by a blinded radiographic review panel composed of the investigator (treating orthopedic spine surgeon) and two reviewers blinded to the device status: a musculoskeletal radiologist and an orthopedic spine surgeon.” |
|  |  | Rationale | Outcome assessors were completely blinded to intervention received. As per the Cochrane handbook, blinded outcome assessment is a low risk of bias. |
| Incomplete outcome data (attrition bias)  ~ fusion rates | High Risk | Direct evidence | “Of the 243 patients, 201 patients completed the study (83%)… Intent-to-treat analysis considered the withdrawn patients to represent failure.” |
|  |  | Rationale | There was a high attrition rate almost balanced between groups. However, the reasons for missing outcomes reported by authors differ, and therefore the heterogeneity may introduce bias. |
| Selective reporting (reporting bias) | Low Risk | Direct evidence | “For the 201-patient evaluable population, repeated-measures analysis of fusion outcomes by GEE ([Figure 4](http://ovidsp.tx.ovid.com.libaccess.lib.mcmaster.ca/sp-3.28.0a/ovidweb.cgi?QS2=434f4e1a73d37e8cdd8fbc96f8f18d4b0b77bb4c6291058d658c16b2a85a1b4036f5c068065561a37d021c607a7e9e5a37da3d6bb904e0e7ebe775d7ac546b4bf84bf82e4603729d03c5a0e2c105aae687a57be4afbce8ad87230562a353eb3e73fcf1ae9287c7e8264df2d3f69d235b82aa618a5ee1d4de534955d67453ffaba0470f04f7b650a4c4a928d21337955e35605ad1b7829265ed56d823e0c9a60f28a2b7b337c043b751689c84b5e1c74c28bc6e315763d1c8ff5936041fef4d7f0c9a00bf246e5a935f251a49bc3568039a67a50d78845737d848240f463650f73f381d7d6b9d34fa884e2c3e3c0dc6715ecc5bb5661a67d131eee86e9c0161d4a8317f51a796b94b8731515b0f44fa762b500ab2034938ecaf024f31b3725e20776d0e25d5190a55387bfe2dc8472d6cd38eefaef69d675bf7b4e95aeb4d257550bb9c9beef500882d89f61bd7ab09b8ca76d9c6d04cc7c95cb2ce4cac46c99e637a2dc7dde0c594d8910b2c40ef1523e27dc4bc4ba0ccc78e96872ebe6daca1595b9d8655c26b46c116ba62bf1cc436a22d24847077b42cb227655c1780855d37ddd4e9f30c7023afb97e61e37e7497585d073c66146c213a8b948eb6c36d19d5782956ba3e2dae1b6251153195d4948ad4bdc85c9f2055d8270dddd9e4fe3f359bf44e8bbab845#FF4)) demonstrated a main effect of treatment, favoring the active treatment (P = 0.030) and, in a separate model, a significant time by treatment interaction (P = 0.024), indicating acceleration of healing.” |
|  |  | Rationale | All outcomes for all patients where reported in strenuous detail. |

**Table 3:** Baseline characteristics of included trials

| **Lead Author** | **Year** | **Country** | **Funding** | **Experimental Group** | | | | **Control Group** | | | | **Outcomes reported** | **Follow-up** |
| --- | --- | --- | --- | --- | --- | --- | --- | --- | --- | --- | --- | --- | --- |
|  |  |  |  | **Mean age  (years)** | **% Males** | **n** | **Lost**  **/missing data** | **Mean age  (years)** | **% Males** | **n** | **Lost**  **/missing data** |  |  |
| Andersen | 2000 | Denmark | Corporate,  Industry &  Federal | 68.9 | 38.1 | 44 | 6 | 71.5 | 31.0 | 33 | 4 | Radiographic fusion rate, Dallas Pain Questionnaire, SF-36, Low Back Pain Rating Scale, walking distance | 24 months |
| Foley | 2008 | U.S.A | None | 46.9 | 55.2 | 122 | 41 | 46.7 | 53.1 | 118 | 42 | Radiographic fusion rate, Mean visual analog scale, mean neck disability  index, SF-12 physical health mean score | 12 months |
| Goodwin | 1999 | U.S.A | Bioelectron Inc. | 45 | 56.5 | 85 | 79 | 40.0 | 52.1 | 94 | 79 | Radiographic & clinical fusion rate | 12 months |
| Jenis | 2000 | U.S.A | N.R | 53.0 (PEMF) 51.0 (DC) | 50.0 (PEMF) 41.2 (DC) | 22 (PEMF) 17 (DC) | 0 | 47.1 | 63.6 | 22 | 0 | Radiographic fusion grade, fusion mass bone density | 12 months |
| Kane | 1988 | U.S.A | N.R | N.R | N.R | 31 | N.R | N.R | N.R | 28 | N.R | Radiographic fusion rate | 18 months |
| Linovitz | 2002 | U.S.A | Corporate &  Industry | 56.77 | 40.8 | 97 | 21 | 56.6 | 36.4 | 104 | 21 | Radiographic fusion rate | 9 months |
| Mooney | 1999 | U.S.A | N.R | 37.9 | 55.1 | 98 | 9 | 37.6 | 52.5 | 97 | 2 | Radiographic fusion rate | 12 months |

**Table 4:** Details of electrical stimulation and control arms with odds ratio of fusion rate.

| **Lead Author** | **Date** | **Type of  stimulation** | **Company name** | **Stimulator frequency (Hz), amplitude, other technical details** | **Treatment Frequency (hrs / day)** | **Treatment Duration** | **Treatment Fusion Rate (%)** | **Control details** | **Control**  **Fusion Rate (%)** | **OR of Fusion Success Rate**  **(Overall)** | **Change in Fusion Rate (Treatment – Control)**  **(%)** |
| --- | --- | --- | --- | --- | --- | --- | --- | --- | --- | --- | --- |
| Andersen | 2009 | DC | Biomet Spine SpF-XL 11b Spine Fusion Simulator | 40 µA and 100 µA | 24 | 6 months – 1 year after primary operation | 64.3% | Dummy electrodes, identical | 57.1% | 1.35 (0.56, 3.25) | 7.2% |
| Foley | 2008 | PEMF | Cervical-Stim®  Osteogenesis  Stimulator | N.R | 4 | 3 months | 83.6% | Inactive sham device | 68.6% | 2.33 (1.26, 4.32) | 15% |
| Goodwin | 1999 | CC | SpinalPak from Biolectron, Inc. | 60kHz delivered via hydrogel surface electrodes | 24 | 9 months | 84.7% | Inactive sham device | 64.9% | 3.00 (1.45, 6.20) | 20% |
| Jenis | 2000 | PEMF DC | PEMF - SpinalStim model 8212 DC - SpF2T stimulator | PEMF - Coil leads placed superficially over fusion site  DC - N.R | PEMF - 2  DC - N.R | PEMF - 5 months  DC - 5 months | 97.4% | Control | 95.5% | 1.81 (0.11, 30.44) | 1.9% |
| Kane | 1988 | DC | Osteostim HS11 | 5µA at each of the four electrodes | N.R | 22 weeks | 80.6% | No implanted stimulator | 53.6% | 3.61 (1.13, 11.52) | 27% |
| Linovitz | 2002 | PEMF | SpinaLogic, OrthoLogic, Tempe, AZ | Single coil worn posteriorly over fusion site | 0.5 | 9 months | 64.4% | Inactive sham device | 43.3% | 2.37 (1.34, 4.18) | 21% |
| Mooney | 1999 | PEMF | Custom design stimulator (based on testing on rabbits) | Brace with multiple coils, 1.5 Hz, 1.8 G magnetic field | 8 | Until healed (although not specifically reported) | 92.2% | Inactive sham device | 67.9% | 5.57 (1.89, 16.41) | 24% |

**Table 5:** Results of the first sensitivity analysis on the basis of incomplete outcome data.

| Analysis | **OR (95% CI), p-value** |
| --- | --- |
| ***Stimulation type*** |  |
| PEMF stimulation | OR = 2.89 (1.50, 5.56), p = 0.001 |
| ***Smoking status*** |  |
| Smokers | OR = 3.20 (1.54, 6.63), p = 0.002 |
| Non-smokers | OR = 2.97 (1.55, 5.00), p = 0.0006 |
| ***Fusion level*** |  |
| Single | OR = 4.03 (1.59, 10.18), p = 0.003 |
| Multiple | OR = 2.79 (1.55, 5.00), p = 0.0006 |
| ***Overall effect*** |  |
| Overall | OR = 2.50 (1.57, 3.98), p = 0.0001 |

**Table 6:** Results of the second sensitivity analysis on the basis of outcome assessment.

| Analysis | **OR (95% CI), p-value** |
| --- | --- |
| ***Stimulation type*** |  |
| DC stimulation | OR = 3.61 (1.13, 11.5), p = 0.03 |
| ***Smoking status*** |  |
| Smokers | OR = 2.87 (1.63, 5.07), p = 0.0003 |
| Non-smokers | OR = 2.42 (1.30, 4.49), p = 0.005 |
| ***Overall effect*** |  |
| Overall | OR = 2.77 (1.99, 3.85), p < 0.00001 |

**Table 7:** Results of the second sensitivity analysis on the basis of outcome assessment.

| Analysis | **OR (95% CI), p-value** |
| --- | --- |
| ***Stimulation type*** |  |
| 1.1.1 PEMF stimulation | OR = 2.89 (1.50, 5.56), p = 0.001 |
| ***Smoking status*** |  |
| 2.1.1 Smoking | OR = 2.76 (1.54, 4.93), p = 0.0006 |
| ***Fusion level*** |  |
| 3.1.1 Single level | OR = 4.03 (1.59, 10.18), p = 0.003 |
| 3.1.2 Multiple levels | OR = 2.79 (1.55, 5.00), p = 0.0006 |
| ***Overall effect*** |  |
| Overall | OR = 2.59 (1.80, 3.73), p < 0.00001 |

**Table 8:** GRADE assessment

| **GRADE domains** | **Rating**  (circle one) | **Footnotes**  (explain reasons for downgrading) | **Quality of evidence**  (Circle one) |
| --- | --- | --- | --- |
| **Outcome: Fusion rates (%)** | | | |
| **Study Design**  (RCT, observational) | High  Low | All studies assessing fusion rates were randomized controlled trials. |  |
| **Risk of Bias**  *(use the Risk of Bias tables and figures)* | Not serious  serious (-1)  very serious (-2) | Three studies had high risk and three studies had unclear risk for allocation concealment. Two studies (Goodwin et al. & Linovitz et al.) had high risk of bias in the domain of incomplete outcome data. A sensitivity analysis  removing these studies resulted in a slight decrease to the pooled effect size (OR = 2.50). | **⊕⊕⊕⊕**  High    **⊕⊕⊕⊖**  Moderate    **⊕⊕⊖⊖**  Low    **⊕⊖⊖⊖**  Very Low |
| **Inconsistency** | Not serious  serious (-1)  very serious (-2) | There is a small amount of variation in the effect size, with confidence intervals overlapping for all 5 studies. Heterogeneity is non-statistically significant (p = 0.57), with an I^2^ value of 0.00%. |  |
| **Indirectness** | Not serious  serious (-1)  very serious (-2) | All studies address the population of interest, which is adult patients (>18 years) undergoing spinal fusion for any spinal pathology. All interventions used in the studies are forms of electrical stimulation (capacitive coupling, direct current, pulsed electromagnetic fields). All studies compare their results to a control intervention, be it placebo or sham. The outcome of fusion rates is reported across all studies. However, outcomes of pain and function are only reported in 2 trials, precluding meta-analysis. Furthermore, all but one studies had a follow-up of at least 12 months or more. Hence, with some limitations, the studies directly address the review question. |  |
| **Imprecision** | Not serious  serious (-1)  very serious (-2) | The total number of events (successful fusions) is 670, which is above the optimal information size (OIS) outlined by Cochrane. Additionally, our total sample size is higher than the calculated OIS. Furthermore, the upper and lower limit of the confidence interval and pooled effect do not include both meaningful benefit and harm, and fall only in meaningful benefit. |  |
| **Publication Bias** | Undetected  Strongly suspected (-1) | GRADE for narrative approach was adopted for this domain, considering a funnel plot was not produced as only 7 studies were included. We do not strongly suspect publication bias as there is a consistent trend of large positive studies and our search was comprehensive. |  |
| **Other**  (upgrading factors, circle all that apply) | Large effect (+1 or +2)  Dose response (+1)  Plausible confounding that is opposing the effect (+1) | N/A |  |

**Table 9.** GRADE summary of findings table

| **Quality assessment** | | | | | | | | **Number of patients** | | **Effect** | **Quality** |
| --- | --- | --- | --- | --- | --- | --- | --- | --- | --- | --- | --- |
| **# Trials** | **Study design rating** | **Risk of bias** | **Inconsistency** | **Indirectness** | **Imprecision** | **Publication bias** | **Other considerations** | **Treatment** | **Control** | **OR (95% CI)** |  |
| **5** | High  (RCT) | Not Serious | Not serious | serious | Not serious | Undetected | N/A | 487 | 454 | 2.53, (1.86, 3.43) | ⊕⊕⊕⊖  Moderate |

| **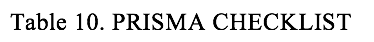Section/topic** | **#** | **Checklist item** | **Reported on page #** |
| --- | --- | --- | --- |
| **TITLE** | | |  |
| Title | 1 | Identify the report as a systematic review, meta-analysis, or both. | 3 |
| **ABSTRACT** | | |  |
| Structured summary | 2 | Provide a structured summary including, as applicable: background; objectives; data sources; study eligibility criteria, participants, and interventions; study appraisal and synthesis methods; results; limitations; conclusions and implications of key findings; systematic review registration number. | 2,3 |
| **INTRODUCTION** | | |  |
| Rationale | 3 | Describe the rationale for the review in the context of what is already known. | 4,5 |
| Objectives | 4 | Provide an explicit statement of questions being addressed with reference to participants, interventions, comparisons, outcomes, and study design (PICOS). | 6 |
| **METHODS** | | |  |
| Protocol and registration | 5 | Indicate if a review protocol exists, if and where it can be accessed (e.g., Web address), and, if available, provide registration information including registration number. | N/A |
| Eligibility criteria | 6 | Specify study characteristics (e.g., PICOS, length of follow-up) and report characteristics (e.g., years considered, language, publication status) used as criteria for eligibility, giving rationale. | 6 |
| Information sources | 7 | Describe all information sources (e.g., databases with dates of coverage, contact with study authors to identify additional studies) in the search and date last searched. | 7,8 |
| Search | 8 | Present full electronic search strategy for at least one database, including any limits used, such that it could be repeated. | 7,8 |
| Study selection | 9 | State the process for selecting studies (i.e., screening, eligibility, included in systematic review, and, if applicable, included in the meta-analysis). | 8 |
| Data collection process | 10 | Describe method of data extraction from reports (e.g., piloted forms, independently, in duplicate) and any processes for obtaining and confirming data from investigators. | 8 |
| Data items | 11 | List and define all variables for which data were sought (e.g., PICOS, funding sources) and any assumptions and simplifications made. | 8 |
| Risk of bias in individual studies | 12 | Describe methods used for assessing risk of bias of individual studies (including specification of whether this was done at the study or outcome level), and how this information is to be used in any data synthesis. | 8,9 |
| Summary measures | 13 | State the principal summary measures (e.g., risk ratio, difference in means). | 9 |
| Synthesis of results | 14 | Describe the methods of handling data and combining results of studies, if done, including measures of consistency (e.g., I^2^) for each meta-analysis. | 9,10 |

| **Section/topic** | **#** | **Checklist item** | **Reported on page #** |
| --- | --- | --- | --- |
| Risk of bias across studies | 15 | Specify any assessment of risk of bias that may affect the cumulative evidence (e.g., publication bias, selective reporting within studies). | 8-10 |
| Additional analyses | 16 | Describe methods of additional analyses (e.g., sensitivity or subgroup analyses, meta-regression), if done, indicating which were pre-specified. | 9,10 |
| **RESULTS** | | |  |
| Study selection | 17 | Give numbers of studies screened, assessed for eligibility, and included in the review, with reasons for exclusions at each stage, ideally with a flow diagram. | 11 |
| Study characteristics | 18 | For each study, present characteristics for which data were extracted (e.g., study size, PICOS, follow-up period) and provide the citations. | 11 |
| Risk of bias within studies | 19 | Present data on risk of bias of each study and, if available, any outcome level assessment (see item 12). | 12 |
| Results of individual studies | 20 | For all outcomes considered (benefits or harms), present, for each study: (a) simple summary data for each intervention group (b) effect estimates and confidence intervals, ideally with a forest plot. | 24 |
| Synthesis of results | 21 | Present results of each meta-analysis done, including confidence intervals and measures of consistency. | 12,13 |
| Risk of bias across studies | 22 | Present results of any assessment of risk of bias across studies (see Item 15). | 12 |
| Additional analysis | 23 | Give results of additional analyses, if done (e.g., sensitivity or subgroup analyses, meta-regression [see Item 16]). | 12,13 |
| **DISCUSSION** | | |  |
| Summary of evidence | 24 | Summarize the main findings including the strength of evidence for each main outcome; consider their relevance to key groups (e.g., healthcare providers, users, and policy makers). | 15-17 |
| Limitations | 25 | Discuss limitations at study and outcome level (e.g., risk of bias), and at review-level (e.g., incomplete retrieval of identified research, reporting bias). | 15-17 |
| Conclusions | 26 | Provide a general interpretation of the results in the context of other evidence, and implications for future research. | 16,17 |
| **FUNDING** | | |  |
| Funding | 27 | Describe sources of funding for the systematic review and other support (e.g., supply of data); role of funders for the systematic review. | 17 |
